# Remodeling of Gut Microbial Networks After Sulforaphane Supplementation in Patients with Chronic Kidney Disease

**DOI:** 10.3390/life15091393

**Published:** 2025-09-02

**Authors:** Marcia Ribeiro, Julie Ann Kemp, Karen Salve Coutinho-Wolino, Ludmila F. M. F. Cardozo, Pedro Almeida, Júnia Schultz, Lia S. Nakao, Maria Eduarda S. Costa, Fernanda Kussi, Henrique F. Santos, Denise Mafra

**Affiliations:** 1 Graduate Program in Biological Sciences—Physiology, Carlos Chagas Filho Institute of Biophysics (IBCCF), Federal University of Rio de Janeiro (UFRJ), Rio de Janeiro 21941-902, RJ, Brazil; marciaribeiro@biof.ufrj.br (M.R.); karenscoutinho@gmail.com (K.S.C.-W.); 2 Graduate Program in Nutrition Sciences, Fluminense Federal University (UFF), Niteroi 24033-900, RJ, Brazil; kempjulie@gmail.com (J.A.K.); ludmilacardozo@id.uff.br (L.F.M.F.C.); 3 Graduate Program in Cardiovascular Sciences, Fluminense Federal University (UFF), Niteroi 24033-900, RJ, Brazil; 4 Department of Marine Biology, Fluminense Federal University (UFF), Niteroi 24033-900, RJ, Brazil; pna.ivo@gmail.com (P.A.); henriquefds@id.uff.br (H.F.S.); 5 Biological and Environmental Science and Engineering Division, King Abdullah University of Science and Technology (KAUST), Thuwal, Makkah 23955, Saudi Arabia; junia.schultz@kaust.edu.sa; 6 Department of Basic Pathology, Federal University of Paraná, Curitiba 81531-980, PR, Brazil; lia.nakao@ufpr.br (L.S.N.); mariacosta@ufpr.br (M.E.S.C.); fernandakussi@ufpr.br (F.K.)

**Keywords:** chronic kidney disease, sulforaphane, gut microbiota, uremic toxins, inflammation, microbial modulation

## Abstract

Background and Objectives: Chronic kidney disease (CKD) is closely associated with gut dysbiosis, and sulforaphane (SFN), a bioactive compound found in cruciferous vegetables, may help to mitigate this condition. Methods: These are secondary exploratory analyses from a previous study that included 16 patients with CKD (stages 3 to 5). The patients were divided into two groups: the Sulforaphane (SFN) group (400 mcg/day of SFN) and the placebo group, both of which received treatment for four weeks. Fecal DNA extraction was performed, and amplicon sequencing was conducted on an Illumina MiSeq V3 platform. The sequence data were analyzed using the QIIME 2 software package. Plasma uremic toxin concentrations (indoxyl sulfate, IS, and p-cresyl sulfate, pCS) were measured by HPLC with fluorescence detection. Results: No significant differences were observed in the gut microbiota alpha microbial richness and diversity after supplementation. However, supplementation with SFN altered the taxonomic composition and resulted in changes to the complexity of the microbial network. A distinct set of Amplicon Sequence Variants (ASVs) was observed post-supplementation with SFN, dominated by genera such as *Megamonas*, *Sphingobium*, *Clostridia*, and *Hungatella*, indicating a treatment-specific microbial signature. The placebo group showed significant increases in IS and pCS, whereas the SFN group presented non-significant changes. Conclusions: SFN supplementation for one month did not significantly alter microbial diversity or uremic toxin levels in non-dialysis CKD patients; however, it led to changes in microbial composition and network complexity, suggesting a modulatory effect on specific microbial interactions.

## 1. Introduction

Chronic kidney disease (CKD), as defined by the National Kidney Foundation Kidney Disease Outcomes Quality Initiative, is characterized by the presence of renal damage identified during clinical evaluation, which includes albuminuria or a glomerular filtration rate that is less than 60 mL/min/1.73 m^2^, persisting for at least 3 months [1]. Patients with CKD face a significant risk of cardiovascular events, experience chronic low-grade inflammation from the early stages of the disease, and exhibit the presence of oxidative stress [2]. Furthermore, researchers have demonstrated that patients with CKD exhibit intestinal dysbiosis, which is closely related to the production of uremic metabolites, accompanied by impaired elimination of these toxins due to kidney function failure [3].

The gut microbiota is a complex and diverse community of trillions of microorganisms that coexist and are involved with the host’s health, playing crucial roles in energy and immune homeostasis. Its composition begins to take shape at birth and can be influenced by various factors, including environmental conditions, lifestyle choices, diet, and concurrent diseases [4]. On the opposite side, intestinal dysbiosis can be understood as a quantitative and qualitative imbalance in the composition and activity of the intestinal microbiota [4,5]. Modulation of the intestinal microbiota in CKD through dietary modifications or supplementation with bioactive compounds has been encouraged as a promising therapeutic approach [6,7].

Sulforaphane (SFN) is the primary bioactive compound found in cruciferous vegetables, produced through the hydrolysis of glucoraphanin by myrosinase. It can help treat numerous chronic diseases, such as diabetes and obesity, and also has antimicrobial, anti-inflammatory, and antioxidant properties [8]. Moreover, recent research has supported the exploration of the role of SFN in modulating gut microbiota [8,9,10,11,12]. Therefore, SFN may have beneficial effects on the intestinal microbiota in patients with CKD. This study aimed to assess the impact of SFN on the intestinal microbiota in non-dialysis CKD patients.

## 2. Materials and Methods

### 2.1. Subjects

This study presents a secondary analysis of a previously published longitudinal clinical trial [13]. This study included 16 patients with CKD stages 3 to 5 who were treated at the Renal Nutrition Outpatient Clinic of the Federal Fluminense University, Niterói, RJ, Brazil. These patients had been undergoing nutritional monitoring for at least six months before the intervention. The inclusion and exclusion criteria were presented in the previous study [13]. The current study was approved by the Ethics Committee of the Faculty of Medicine at UFF (number 39904520.8.0000.5243) and registered with ClinicalTrials.gov (NCT04608903).

### 2.2. Experimental Design

As described previously in our study, patients selected according to the eligibility criteria were allocated to the sulforaphane (SFN) or the placebo group. Blinding of the supplements was carried out by a pharmacist, as described in Ribeiro et al. (2024) [13]. Patients allocated to the SFN group were instructed to consume two capsules of 200 mcg of L-sulforaphane (i.e., 400 mcg/day of L-sulforaphane) daily, including weekends, for one month, to provide approximately 150 μmol of SFN, as per a previous study [14]. The amount of SFN in the capsules was determined by HPLC with UV detection, as described in the previous study [13]. Likewise, patients allocated to the placebo group were instructed to consume two capsules containing 200 mcg of corn starch. Telephone contact was made during the study period to encourage the consumption of the capsules and record reports of possible side effects. The remaining capsules from both groups were collected on the day of the second collection and counted to calculate the adhesion percentage.

For the analysis, stool and blood samples were collected before and after the supplementation period. The blood was processed to obtain serum and plasma for subsequent analysis. Food intake was assessed at the beginning and end of the intervention using a three-day food record technique, which covered two weekdays and one weekend day. As presented in the previous study, the energy and macronutrient intake analysis were estimated using the DietBox^®^ software [13].

### 2.3. DNA Extraction and Sequencing

Patients received a specific sterile container for collecting stools and were provided with instructions on how to collect the samples. They were instructed to keep the samples in the freezer overnight and deliver them the next day to the collection site. Stool samples were taken to the laboratory and stored at −20 °C for future analysis. DNA extraction was performed on sixteen samples using the Quick-DNA Fecal/Soil Microbe DNA Miniprep Kit (Zymo Research, Irvine, CA, USA) according to the manufacturer’s instructions. To determine the final DNA concentrations, spectrophotometric quantification was conducted using NanoDrop 2000 (Thermo Fisher Scientific, Waltham, MA, USA).

Subsequently, DNA purity and concentration were assessed using a Qubit fluorometer (Thermo Fisher Scientific, Waltham, MA, USA). Samples were sent to StarSEQ GmbH (Mainz, Germany) for amplification and sequencing of the V4/V5 region of the 16S ribosomal RNA (rRNA) gene. PCR reactions employed the primers 515F (GTGYCAGCMGCCGCGGTAA) and 909R (CCCCGYCAATTCMTTTRAGT), using the same thermal cycling conditions described by [15]. Amplicon sequencing was conducted on an Illumina MiSeq V3 platform (Illumina, San Diego, CA, USA) using a single-end library.

### 2.4. Gut Microbiota Analysis

The QIIME 2 software package (version 2017.10) (https://qiime2.org/) (URL accessed in April 2024) was used to process the raw sequence data. The reads were obtained after demultiplexing with q2-DEMUX, followed by quality filtering and removal of chimeras using q2-DADA2 [16]. The DADA2 plugin was employed to evaluate read quality and cluster the reads into Amplicon Sequence Variants (ASVs) [16]. Taxonomic classification of each ASV was performed using the feature-classifier plugin, which utilizes a Bayesian classifier trained on the SILVA 138 database [17]. Singleton sequences, chloroplasts, and mitochondria were removed from the dataset before further analysis.

Alpha diversity analysis, including observed ASVs and Shannon index [18,19], was performed using the R programming language (version 3.6.3) (R Core Team, 2015). For the beta diversity analysis, the sequencing data matrices were sorted using multidimensional scaling (MDS) analysis [20], facilitated by the Phyloseq package [21]. To assess the variations between the different sample types, the Kruskal–Wallis [22] and PERMANOVA tests [23] were applied using R software. Results with *p*-values < 0.05 were considered significant.

To explore the number of unique and shared ASVs among sample groups, an UpSet plot was used in place of the traditional Venn diagram. This visualization approach enables the identification of complex intersection patterns among multiple sets, offering improved scalability and interpretability, particularly for datasets with a large number of groups or ASVs.

To identify potential microbial biomarkers associated with treatment effects, a differential abundance analysis was performed using the Microbiota Process package [24]. Specifically, the mp_diff_analysis function was applied, which employs the Kruskal–Wallis test (for between-group comparisons) and the Wilcoxon signed-rank test (for paired comparisons), with a significance threshold set at α = 0.05. Significant taxonomic differences were visualized through a differential cladogram, generated using the mp_plot_diff_cladogram function [24]. This plot displays the hierarchical clustering of samples based on the relative abundances of ASVs that were differentially abundant between groups. Highlighted clades in the cladogram represent taxa with statistically significant differences and were colored according to the experimental group (before or after; SFN or placebo). The node sizes were scaled according to the Kruskal–Wallis test statistic (FDR-corrected), providing a visual indication of the statistical magnitude of the observed differences. This approach enabled an integrated visualization of phylogenetic structure and differential microbial shifts, facilitating the identification of species with potential functional or clinical relevance following the intervention.

The network construction utilized a set of nodes, each representing a genus of microorganisms. This level was chosen to minimize the introduction of noise into the networks from the ASVs while maintaining relevant taxonomic resolution. Taxonomic levels higher than genus could lead to a loss of resolution. The edges connecting the nodes represented significantly positive or negative correlations between genera. Co-occurrence patterns in bacterial communities were assessed using the R programming language (version 3.6.3) (R Core Team, 2015), with a relative abundance matrix of genera as input data. The SparCC algorithm [25], which estimates Pearson’s correlation values from compositional data, was used for the co-occurrence analysis. Only SparCC correlations with magnitudes greater than 0.4 or less than −0.4 were considered. The SpiecEasi library [26] in R was employed to generate the networks.

### 2.5. Analysis of Uremic Toxins

Total plasma concentrations of indoxyl sulfate (IS) and p-cresyl sulfate (p-CS) were quantified by reversed-phase high-performance liquid chromatography (HPLC) coupled with fluorescence detection. Plasma samples were processed as described by Meet et al., 2012 [27]. Briefly, 100 μL of plasma was diluted with water to a final volume of 360 μL and heated at 95 °C for 30 min. After cooling on ice for 10 min, the samples were centrifuged at 1300× *g* for 20 min at 4 °C. The supernatant obtained was subjected to ultrafiltration through a 30 kDa cutoff membrane (Amicon Ultra, Millipore, Merck, Germany). A 10 μL volume of the ultrafiltrate was injected into the HPLC system.

### 2.6. Statistical Analysis

The Shapiro–Wilk test was utilized to test the normal distribution of the data. The independent *t*-test (for parametric data) or the Mann–Whitney test (for nonparametric data) was used to analyze the results. A chi-squared test was utilized to evaluate the frequency data. The comparison before and after one month of supplementation in each group was performed using the paired-sample *t*-test for parametric data and the Wilcoxon test for non-parametric data, and a two-way ANOVA was used for analysis of variance between groups. Data were expressed as mean and standard deviation, with significance levels set at 5% (*p* < 0.05). Statistical analysis was carried out using SPSS 25.0 software.

## 3. Results

### 3.1. Patients

This study involved 16 non-dialysis patients, 12 of whom were women, with an average age of 61.8 ± 11.6 years, an estimated glomerular filtration rate (eGFR) of 37.4 ± 12.9 mL/min, and a BMI of 31.0 ± 6.6 Kg/m^2^ (Table 1). After the intervention period, these parameters did not change statistically, including eGFR and BMI. Out of the 16 study participants, 15 had systemic arterial hypertension and were on antihypertensive medications. Additionally, 5 of the 16 participants were also taking medication to manage diabetes mellitus. During the intervention, side effects were not reported. No significant changes in food intake were observed between the groups at the beginning of the study, nor after one month of intervention in the SFN group, as reported in the previous study [13]. We calculated an adherence percentage of 96% in the SFN group from the capsules collected after the intervention period.

### 3.2. Structure and Diversity of Gut Microbiota

**Figure 1:**
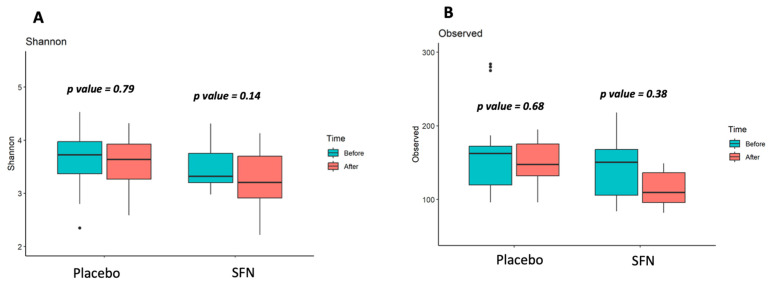
Gut microbiota alpha-diversity of patients with CKD on SFN and placebo supplementation. (**A**) Shannon index (diversity), (**B**) Observed ASVs (richness).

Stool samples from 16 non-dialysis patients were analyzed to establish the gut microbiota community. We compared the gut microbiota of patients grouped with SFN and placebo, and also before and after supplementation, regarding diversity and taxonomic composition. No significant differences were observed after the supplementation period in the gut microbiota alpha microbial richness (observed number of ASVs, Figure 1B) and diversity (Shannon index, Figure 1A).

**Figure 2:**
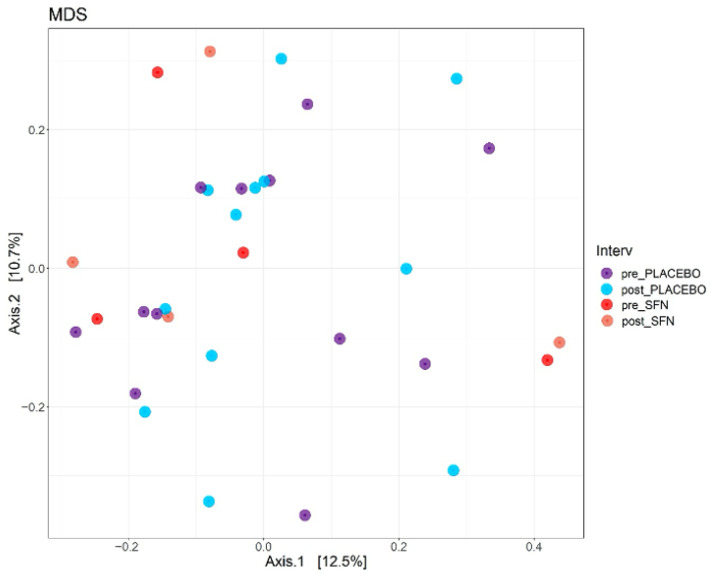
Multidimensional scaling (MDS) plots of the CKD patients in the SFN and placebo groups before and after intervention.

Figure 2 illustrates beta diversity, as determined by a Bray–Curtis-based multidimensional scaling (MDS) analysis of gut microbiota composition in patients with CKD who received a diet supplemented with SFN or placebo for one month. The results revealed a variation of only 12.5% (*p*-value = 0.001) in the bacterial community among individuals, and no statistically significant differences were detected between pre- and post-intervention time points in either the SFN or placebo groups. SFN supplementation did not significantly alter the gut microbiota at the level of bacterial community.

**Figure 3:**
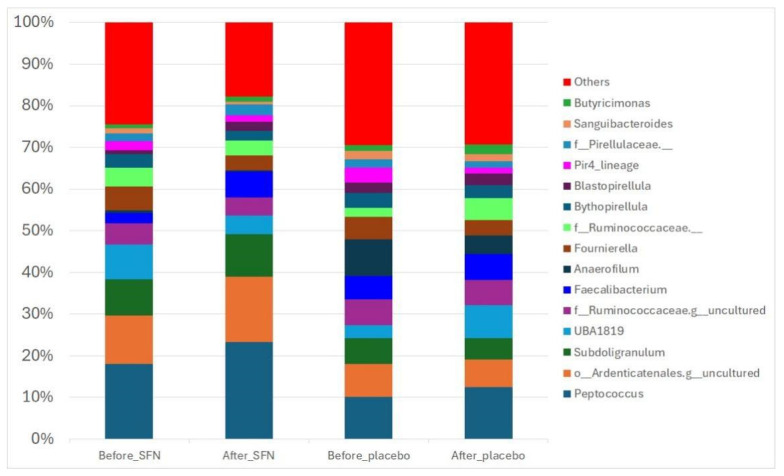
Gut bacterial composition of the SFN and placebo groups before and after the intervention. The compositions are presented as relative abundances at the genus level.

Figure 3 shows the 15 most abundant microbial taxa across the analyzed samples. At the genus level, thirteen genera had a relative abundance accounting for 47% of the total bacteria before SFN supplementation, with *Peptococcus* (18%), *Subdoligranulum* (8%), and *UBA1819* (9%) as the most abundant taxa. After the intervention with SFN, the relative abundance of *Peptococcus* increased to 22%. In contrast, the relative abundance of *UBA1819* decreased.

**Figure 4:**
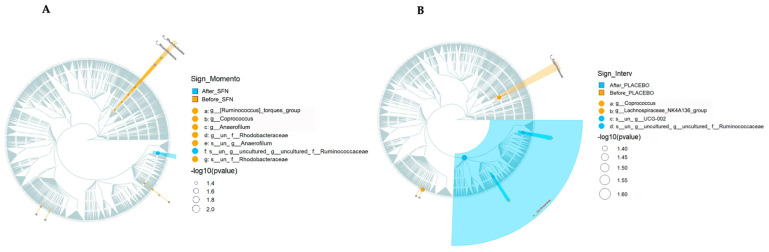
Cladogram representation of microbiome taxa associated with SFN or placebo. The treatments evaluated were (**A**) SFN and (**B**) placebo. Yellow indicates taxa enriched in the pre-treatment period, and blue indicates taxa enriched in the post-treatment period. Only taxa with nominal *p* < 0.05 are labeled.

Cladogram analysis (Figure 4A, SFN group, and 4B, placebo group) was conducted to identify ASVs that exhibited statistically significant differences (*p* < 0.05) in abundance before and after each treatment. It can be observed that before supplementation with SFN, the most predominant ASVs were *Rhodobacteraceae* and *Rhodobacterales*. In the placebo group, the most predominant ASV before treatment was *Eggerthellaceae*. In both groups, the most predominant ASV after treatment was *Ruminococcaceae*.

**Figure 5:**
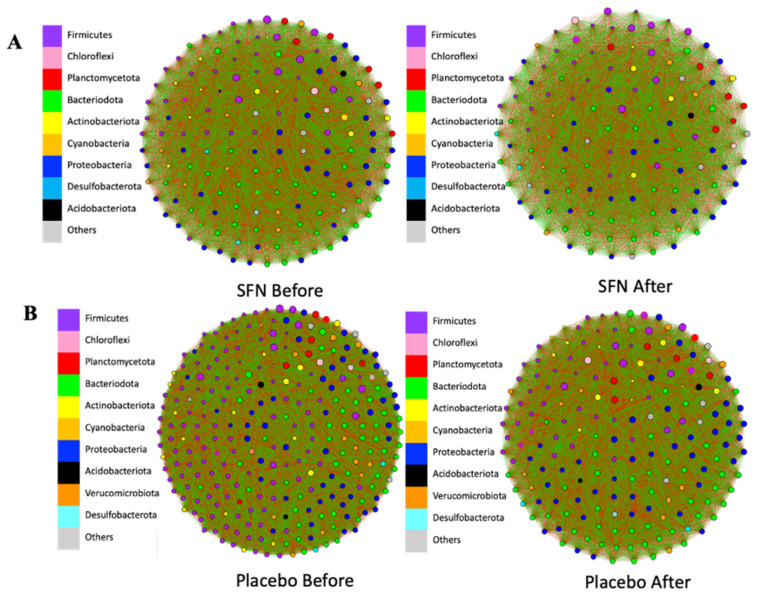
Gut bacterial co-occurrence networks at the genus level in the SFN group (**A**) and placebo group (**B**) before and after the intervention. Green: positive association; red: negative association. Node size represents the relative abundance of the genera in their respective phylum.

The gut bacterial co-occurrence networks were compared between the SFN and placebo groups (Table S1). Figure 5 shows the co-occurrence networks of colored genera in phyla of the SFN and placebo groups before and after the interventions. After one month of intervention with SFN, it was possible to observe a change in the central genera of the connections in the SFN group. The number of nodes (individual taxa) and edges (green lines represent positive connections between genera, while the red lines represent negative connections) decreased at most taxonomic levels. Furthermore, at the genus level, the total number of edges in the SFN group before intervention was 11,781, and it reduced to 7750 after SFN; that is, the microbial complexity of this group changed by 33%. The positive edges reduced from 5487 to 3679, and the negative from 6294 to 4071 in the SFN group (Figure 5A). In contrast, the placebo group exhibited even more pronounced changes; the total number of edges in the placebo group before the intervention was 40,186, which decreased to 18,145, representing a 54.8% reduction. The positive edges reduced from 18,428 to 8217, and the negative from 21,728 to 9928 in this group (Figure 5B).

**Figure 6:**
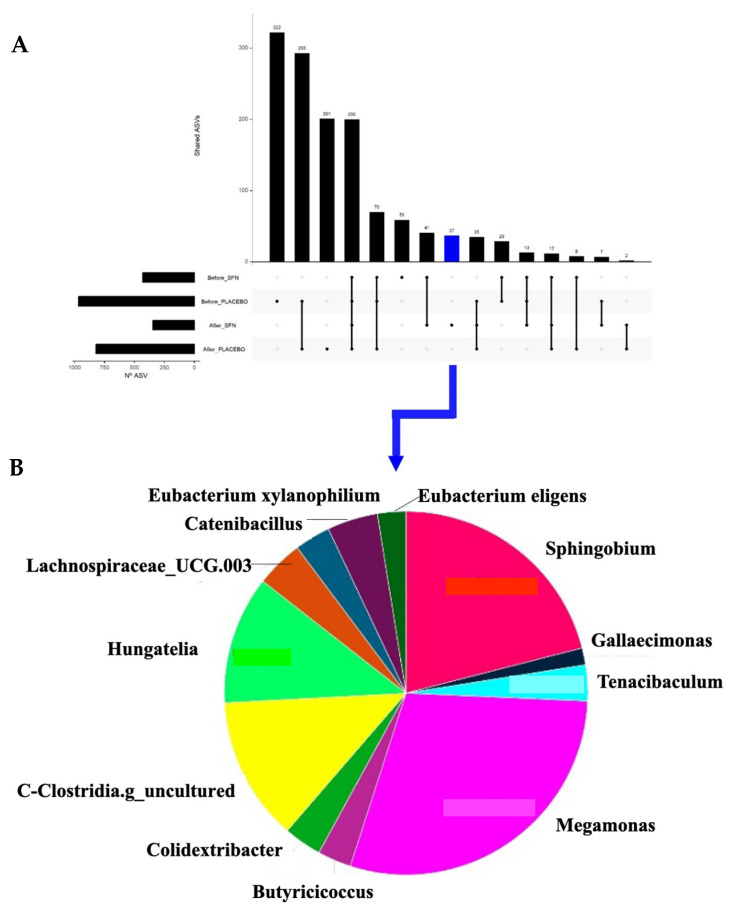
Shared ASVs across time and groups of intervention. (**A**) UpSet plot showing the number of shared and unique ASVs among the intervention groups. (**B**) Pie chart of the unique ASVs found in the gut microbiota of patients after intervention with SFN.

A total of 200 ASVs were shared between all four groups (SFN and placebo before and after), indicating a core microbiota (Figure 6). Several group-specific features are also highlighted, suggesting that specific microbial taxa are either unique to or enriched in certain conditions (e.g., SFN vs. placebo, or before and after intervention). Notably, 37 ASVs were exclusively observed after SFN supplementation (blue, Figure 6A). The presence of a distinct set of ASVs post-supplementation with SFN (Figure 6B) and their composition, dominated by genera such as *Megamonas*, *Sphingobium*, *Clostridia*, and *Hungatella*, indicates a treatment-specific microbial signature. This may point to potentially beneficial or functionally relevant changes induced by SFN.

### 3.3. Uremic Toxins

The placebo group showed significant increases in IS and pCS after the intervention. In contrast, the SFN group exhibited non-significant increases in both toxins, which may suggest a protective trend, albeit with a small sample size (Table 2). It was observed that pCS and IS correlated positively with the genera *Subdoligranulum*, *UBA1819*, *Anaerophilium*, and *Fournierella*.

## 4. Discussion

This study aimed to evaluate the effects of SFN on the intestinal microbiota composition in non-dialysis CKD patients. After one month of intervention with SFN, no significant changes were observed in the diversity of the intestinal microbiota. However, supplementation with sulforaphane altered the taxonomic composition and resulted in changes to the complexity of the microbial network.

To our knowledge, this is the first study to document the effects of SFN on gut microbiota diversity in patients with CKD. In experimental models, several studies have shown promising effects of SFN in modulating gut microbiota diversity. Wang et al. [9] found an increase in beta diversity of gut microbiota after treatment with 10 mg/kg of SFN for 6 weeks in hyperuricemic rats. Additionally, Jun et al. [28] demonstrated that SFN supplementation at a concentration of 442.5 mg/kg for 2 months improved both the diversity and richness of the gut microbiota in aged mice. Another study demonstrated that the intake of broccoli seed extract, rich in glucoraphanin (a precursor of SFN), led to an increase in the diversity of the gut microbiota in mice with acute liver damage [10].

Although these experimental results are favorable, clinical trials are still quite limited, which makes it difficult to draw solid conclusions about the effect of SFN on gut microbiota diversity. Other clinical trials using different types of bioactive compounds, both in the short term (3 days) [29] or in the long term (6 weeks) [30], have shown no significant differences in the gut microbiota diversity, indicating that the lack of difference may not be associated with the time of intervention, but may be with the dosage used. Moreover, it is essential to note that gut microbiota composition and consequently its diversity are influenced by several factors [31]. Indeed, changes in physiology, diet, medication, and lifestyle can affect the diversity of the gut microbiota [28]. Although no significant diversity results were found in this study, interesting results were observed regarding the taxonomic composition.

As shown in the results mentioned above, SFN supplementation modulated the taxonomic composition of the intestinal microbiota of the study participants. A reduction in the abundance of the genus UBA1819 was observed, which, according to the literature, may be beneficial to patients. Qin et al. (2025) [32] observed that patients undergoing hemodialysis have an increased abundance of the genus UBA1819, which was associated with renal complications, including anemia. Corroborating these results, Chen et al. (2022) [33] also demonstrated that patients with diabetes and CKD had an increased abundance of the genus UBA1819, and this abundance was positively correlated with the estimated glomerular filtration rate. Thus, the reduction observed in this study may be beneficial to the patients studied. Similar to our result, Zhang et al. (2023) [34] supplemented 200 mg/kg of extract from a traditional medicinal plant rich in flavanols, called Epimedium, in birds and observed a reduction in harmful bacteria, such as UBA1819. According to the authors, a reduction in this pathogenic genus implies an improvement in the intestinal microbiota balance, resulting in healthier microbiota.

Among the observed modulations, the increase in the Peptococcus genus stands out. Previous studies investigating the regulation of plasma cholesterol in hypercholesterolemic hamsters [35] and the regulation of the Nrf-2/HO-1 signaling pathways [36] underscore its clinical significance. Peptococcus is an anaerobic gram-positive cocci bacterial genus that negatively correlates with the production of short-chain fatty acids (SCFAs), essential fermentation products of the human microbiota associated with cholesterol regulation and inflammation [35,37]. Furthermore, Yan et al. (2024) [36] observed, in their experimental investigation of the use of a polysaccharide widely used in Chinese medicine for treating non-alcoholic fatty liver disease in rats for 6 weeks, an increase in the abundance of Peptococcus. Also, they demonstrated that the genus Peptococcus correlated negatively with indicators of oxidative stress, such as malondialdehyde, and positively with markers of inflammation, including SOD-2, CPT1α, and PPARα [36]. An experimental model of CKD confirms the presence of the genus Peptococcus in the CKD rat model induced by adenine at 150 mg/kg [38].

A predominance of the *Ruminococcaceae* genus was observed in both the placebo and sulforaphane groups after the intervention period, as shown in the cladogram. According to studies by Xu et al. (2017) [39] and Gryp et al. (2017) [40], this predominance may be beneficial to patients with CKD, who naturally present with reduced percentages of this beneficial microbial genus. *Ruminococcaceae* are beneficial genera that produce short-chain fatty acids, and a reduced rate of this genus may contribute to uremic toxicity, inflammation, and clinical worsening in patients with CKD [41,42]. Thus, although this was not an isolated effect of sulforaphane, our study observed an increase in the percentage of the *Ruminococcaceae* genus in the evaluated patients with CKD, corroborating their clinical improvement.

Regarding gut microbiota co-occurrence, interest in this analysis is emerging due to the exploration of microbial interactions and their stability in the environment beyond compositional aspects [43]. Additionally, gut microbial stability has been linked to host health [44]. In addition to the stability, some studies have highlighted that a high modularity, lower positive interactions, and higher negative interactions lead to a more robust and stable gut microbial community [44,45,46]. In our study, we observed that the SFN group, after the intervention period, reduced their network complexity, total edges, and both positive and negative associations. In comparison, the placebo also exhibited a reduction in the same co-occurrence items, albeit to a lesser extent. We suggest that the gut microbiota, in response to SFN intervention, may reorganize itself, reduce complexity, and possibly become more stable compared to the placebo. A lower network complexity may be associated with more well-defined and cohesive modules, which can lead to a microbial community structure by functional group.

Sun et al. (2023) [44] evaluated the co-occurrence networking in the gut microbiota of mice after four weeks on a high-fat diet. The authors observed that this unhealthy diet increased the complexity of the gut microbial network and positive associations, while decreasing the modularity. The authors described this environment as more unstable [44]. Positive associations within the gut microbial network may respond more to environmental changes within the group, which can amplify the disturbance [45].

Concerning uremic toxins and gut microbiota in CKD, Gryp et al. (2020) reported 92 bacterial species that were protein-bound uremic toxins (PBUT) precursor-generators [47]. However, none of the bacteria found in our study were described by Gryp et al. (2020) as producing a PBUT [47]. Despite the limited number of samples, an increase in plasma uremic toxin levels was observed in the placebo group, reinforcing the biological relevance of our findings.

Furthermore, a unique microbial signature was observed after sulforaphane supplementation, in which the genera Megamonas, Sphingobium, Clostridia, and Hungatella were observed. Understanding whether this unique signature could reflect the functional modulation of the gut microbiota induced by SFN, with enhanced metabolic or anti-inflammatory potential, for example, is not straightforward. When investigating the genus Megamonas, it is observed that this genus is associated with the production of SCFAs, especially acetate and propionate [48,49]. SCFAs are the primary metabolites produced in the colon through the fermentation of dietary fiber by bacteria. They are associated with trophic factors in the intestine, as well as with neurological, immune, and endocrine regulation [50]. Studies have shown that Megamonas seems to be increased in healthy individuals [51] and reduced in patients with CKD [52].

Regarding the genus Sphingobium, further research is necessary, as no studies have been conducted on it. The genus Clostridia, belonging to the phylum Firmicutes, was also observed in this unique microbial signature. Unlike what we observed in our study, Huang et al. (2021) [53] found a reduced abundance of this genus in patients with diabetes (with or without retinopathy) compared to healthy individuals. A reduction in these beneficial bacteria in diabetic patients is detrimental [54,55]. Therefore, the occurrence of this genus in our study may be helpful for patients with CKD. Finally, the genus Hungatella completes the unique microbial signature observed in this study after SFN supplementation. Some bacterial genera are associated with tumors; in the case of Hungatella, it has been seen in the literature that this genus could contribute to tumor regression through modifications in metabolism and immune response [56,57]. This action is consistent with the salutary implications associated with SFN found in the literature. Sulforaphane has been associated with anticancer action due to its ability to interact with several signaling pathways involved in the growth and survival of cancer cells [58]. The relevance of the intestinal microbiota and cancer axis has drawn increasing attention not only for its detection, but also in cancer therapy.

Furthermore, this study found a positive correlation between the pCS toxin and the UBA1819 genus, as well as a positive correlation between the IS and the Anaerofilum genus. In this respect, it has been demonstrated that the UBA1819 genus is associated with multiple sclerosis, and the Anaerofilum genus is linked to obesity, characterized as a bacterium present in the obesogenic microbiota [59,60]. Interestingly, pCS induces neurotoxicity in multiple sclerosis, and the IS is associated with several obesity parameters, including waist-to-hip ratio and conicity index [61,62]. These data suggest that the presence of such bacteria and their correlation with uremic toxins may be indicative of adverse clinical effects in these patients. Also, uremic toxins were positively correlated with family Ruminococcaceae, which includes the genera Subdoligranulum and Fournierella. These correlations were expected as these bacteria have been linked to patients with acute ischemic stroke with preceding infection [63], heart failure [64], and several studies show that such uremic toxins are associated with adverse cardiovascular events by inducing inflammation, fibrosis, left ventricular hypertrophy, and promoting peripheral artery disease in CKD [65,66].

The clinical relevance of this study is evident in the observed changes in the composition and co-occurrence network of the gut microbiota in non-dialysis CKD patients. The modulation of genera associated with adverse clinical outcomes in CKD patients, such as the reduction in *UBA1819* and the emergence of a unique microbial signature after sulforaphane supplementation (including *Megamonas* and *Clostridia*), suggests a possible functional reprogramming of the microbiota with anti-inflammatory and metabolic implications. Furthermore, the correlation between specific bacterial genera and uremic toxins known for their nephrotoxicity and association with cardiovascular comorbidities highlights the potential of sulforaphane as a therapeutic modulator of the gut–kidney axis interaction. These findings reinforce the importance of nutritional interventions based on bioactive compounds as adjunctive strategies in the clinical management of CKD, highlighting the need for future studies with larger sample sizes and longer intervention times.

This study has many limitations. The short intervention period and the dosage used in this study may not have been sufficient to induce more pronounced effects on the gut microbiota. Also, the small sample may compromise the strength of the statistical analyses and the robustness of the conclusions. We also point out that not analyzing food consumption and its correlation with the results of this study is a limitation. Additionally, the gut microbiota of patients with CKD may be less responsive to modulation due to existing dysbiosis and uremic conditions. On the other hand, this is one of the first studies to investigate the effect of SFN supplementation on gut microbiota in patients with CKD, offering valuable insights into potential microbial shifts.

## 5. Conclusions

Although a one-month intervention with 400 mcg/day of SFN did not significantly alter microbial diversity in non-dialysis CKD patients, changes in the taxonomic composition and microbial network complexity were observed. These findings suggest that SFN supplementation may modulate specific microbial interactions rather than general diversity indices. Additionally, while the placebo group showed significant increases in uremic toxin levels, the SFN group experienced only non-significant changes, suggesting a potential protective trend. However, the effects of SFN on the gut microbiota of patients with CKD remain poorly understood, and further studies are needed to explore its role, optimal dosage, and duration in this specific population.

## Figures and Tables

**Table 1:** Baseline demographic and clinical characteristics of the enrolled patients.

Parameters	SFN Group (*n* = 4)	Placebo Group (*n* = 12)	*p*-Values
Sex (Female/Male)	4/0	8/4	0.18
Age (years)	60.5 ± 15.1	61.6 ± 10.9	0.86
BMI (kg/m^2^)	28.6 ± 7.0	30.5 ± 7.1	0.59
eGFR (mL/min/1.73 m^2^)	39.2 ± 15.6	36.7 ± 12.6	0.75
Urea (mg/dL)	60.2 ± 17.7	60.2 ± 13.5	1.00
Calcium (mg/dL)	10.3 ± 0.6	10.2 ± 0.4	0.74
Phosphorus (mg/dL)	4.2 ± 0.4	4.3 ± 0.8	0.80
Albumin (g/dL)	4.2 ± 0.1	4.5 ± 0.3	0.05
Potassium (mmol/L)	4.8 ± 0.1	4.7 ± 0.4	0.67

A comparison between groups was carried out using the independent *t*-test, the Mann–Whitney test, and the chi-square test. Data are expressed as mean ± standard deviation. Differences with *p* < 0.05 were considered statistically significant. Abbreviations: BMI: body mass index; eGFR: estimated glomerular filtration rate; SFN: sulforaphane group.

**Table 2:** Uremic toxin levels before and after sulforaphane supplementation.

Uremic Toxins	SFN Group (*n* = 4)					Placebo Group (*n* = 12)			*p*-Value ANOVA
	Before	After	Percentage Change (%)	*p*- Values	Before	After	Percentage Change (%)	*p*- Values	
Indoxyl sulfate (mg/L)	1.7 ± 0.1	2.6 ± 1.3	47.9	0.06	2.1 ± 0.8	3.6 ± 2.5	81.5	**0.04**	0.65
p-cresyl sulfate (mg/L)	5.9 ± 2.4	8.1 ± 6.6	42.1	0.46	8.9 ± 6.7	15.9 ± 12.5	77.4	**0.002**	0.55

Analyses were carried out within groups. Comparison of the data before and after supplementation in each group was performed using the paired-sample *t*-test for parametric data and the Wilcoxon test for non-parametric data. Data are expressed as mean ± SD. *p*-value ANOVA for analysis of variance between groups. Bold values indicate a significant change (*p* < 0.05).

## Data Availability

The original contributions presented in this study are included in the article/Supplementary Materials. Further inquiries can be directed to the corresponding author.

## Supplementary Materials

The following supporting information can be downloaded at: https://www.mdpi.com/article/10.3390/life15091393/s1, Table S1: Co-occurrence networks between the SFN and placebo groups.

## References

[1] Stevens P.E., Ahmed S.B., Carrero J.J., Foster B., Francis A., Hall R.K., Herrington W.G., Hill G., Inker L.A., Kazancıoğlu R. (2024). KDIGO 2024 Clinical Practice Guideline for the Evaluation and Management of Chronic Kidney Disease. Kidney Int..

[2] Jankowski J., Floege J., Fliser D., Böhm M., Marx N. (2021). Cardiovascular Disease in Chronic Kidney Disease Pathophysiological Insights and Therapeutic Options. Circulation.

[3] Rysz J., Franczyk B., Ławiński J., Olszewski R., Ciałkowska-Rysz A., Gluba-Brzózka A. (2021). The Impact of CKD on Uremic Toxins and Gut Microbiota. Toxins.

[4] Gomaa E.Z. (2020). Human Gut Microbiota/Microbiome in Health and Diseases: A Review. Antonie Van Leeuwenhoek Int. J. Gen. Mol. Microbiol..

[5] Chen Y.Y., Chen D.Q., Chen L., Liu J.R., Vaziri N.D., Guo Y., Zhao Y.Y. (2019). Microbiome-Metabolome Reveals the Contribution of Gut-Kidney Axis on Kidney Disease. J. Transl. Med..

[6] Voroneanu L., Burlacu A., Brinza C., Covic A., Balan G.G., Nistor I., Popa C., Hogas S., Covic A. (2023). Gut Microbiota in Chronic Kidney Disease: From Composition to Modulation towards Better Outcomes—A Systematic Review. J. Clin. Med..

[7] Mafra D., Borges N., Alvarenga L., Esgalhado M., Cardozo L., Lindholm B., Stenvinkel P. (2019). Dietary Components That May Influence the Disturbed Gut Microbiota in Chronic Kidney Disease. Nutrients.

[8] Sun Y., Tang Z., Hao T., Qiu Z., Zhang B. (2022). Simulated Digestion and Fermentation In Vitro by Obese Human Gut Microbiota of Sulforaphane from Broccoli Seeds. Foods.

[9] Wang R., Halimulati M., Huang X., Ma Y., Li L., Zhang Z. (2023). Sulforaphane-Driven Reprogramming of Gut Microbiome and Metabolome Ameliorates the Progression of Hyperuricemia. J. Adv. Res..

[10] Mao B., Ren B., Wu J., Tang X., Zhang Q., Zhao J., Zhang L., Chen W., Cui S. (2023). The Protective Effect of Broccoli Seed Extract against Lipopolysaccharide-Induced Acute Liver Injury via Gut Microbiota Modulation and Sulforaphane Production in Mice. Foods.

[11] Bouranis J.A., Beaver L.M., Wong C.P., Choi J., Hamer S., Davis E.W., Brown K.S., Jiang D., Sharpton T.J., Stevens J.F. (2024). Sulforaphane and Sulforaphane-Nitrile Metabolism in Humans Following Broccoli Sprout Consumption: Inter-Individual Variation, Association with Gut Microbiome Composition, and Differential Bioactivity. Mol. Nutr. Food Res..

[12] Yang J., He L., Dai S., Zheng H., Cui X., Ou J., Zhang X. (2023). Therapeutic Efficacy of Sulforaphane in Autism Spectrum Disorders and Its Association with Gut Microbiota: Animal Model and Human Longitudinal Studies. Front. Nutr..

[13] Ribeiro M., Alvarenga L., Coutinho-Wolino K.S., Nakao L.S., Cardozo L.F., Mafra D. (2024). Sulforaphane Upregulates the MRNA Expression of NRF2 and NQO1 in Non-Dialysis Patients with Chronic Kidney Disease. Free Radic. Biol. Med..

[14] Mirmiran P., Bahadoran Z., Hosseinpanah F., Keyzad A., Azizi F. (2012). Effects of Broccoli Sprout with High Sulforaphane Concentration on Inflammatory Markers in Type 2 Diabetic Patients: A Randomized Double-Blind Placebo-Controlled Clinical Trial. J. Funct. Foods.

[15] Parada A.E., Needham D.M., Fuhrman J.A. (2016). Every base matters: Assessing small subunit rRNA primers for marine microbiomes with mock communities, time series and global field samples. Environ. Microbiol..

[16] Callahan B.J., McMurdie P.J., Rosen M.J., Han A.W., Johnson A.J.A., Holmes S.P. (2016). DADA2: High-Resolution Sample Inference from Illumina Amplicon Data. Nat. Methods.

[17] Quast C., Pruesse E., Yilmaz P., Gerken J., Schweer T., Yarza P., Peplies J., Glöckner F.O. (2013). The SILVA Ribosomal RNA Gene Database Project: Improved Data Processing and Web-Based Tools. Nucleic Acids Res..

[18] Ward J.V., Tockner K., Schiemer F. (1999). Biodiversity of Floodplain River Ecosystems: Ecotones and Connectivity1. Regul. Rivers Res. Manag..

[19] Shade A. (2017). Diversity Is the Question, Not the Answer. ISME J..

[20] Wickelmaier F. (2003). An Introduction to MDS. Sound Quality Research Unit.

[21] McMurdie P.J., Holmes S. (2013). Phyloseq: An R Package for Reproducible Interactive Analysis and Graphics of Microbiome Census Data. PLoS ONE.

[22] Kruskal W.H., Wallis W.A. (1952). Use of ranks in one-criterion variance analysis. J. Am. Stat. Assoc..

[23] Kelly B.J., Gross R., Bittinger K., Sherrill-Mix S., Lewis J.D., Collman R.G., Bushman F.D., Li H. (2015). Power and Sample-Size Estimation for Microbiome Studies Using Pairwise Distances and PERMANOVA. Bioinformatics.

[24] Xu S., Zhan L., Tang W., Wang Q., Dai Z., Zhou L., Feng T., Chen M., Wu T., Hu E. (2023). MicrobiotaProcess: A Comprehensive R Package for Deep Mining Microbiome. Innovation.

[25] Friedman J., Alm E.J. (2012). Inferring Correlation Networks from Genomic Survey Data. PLoS Comput. Biol..

[26] Kurtz Z.D., Müller C.L., Miraldi E.R., Littman D.R., Blaser M.J., Bonneau R.A. (2015). Sparse and Compositionally Robust Inference of Microbial Ecological Networks. PLoS Comput. Biol..

[27] Meert N., Schepers E., Glorieux G., Van Landschoot M., Goeman J.L., Waterloos M.A., Dhondt A., Van Der Eycken J., Vanholder R. (2012). Novel Method for Simultaneous Determination of P-Cresylsulphate and p-Cresylglucuronide: Clinical Data and Pathophysiological Implications. Nephrol. Dial. Transplant..

[28] Jun S.R., Cheema A., Bose C., Boerma M., Palade P.T., Carvalho E., Awasthi S., Singh S.P. (2020). Multi-Omic Analysis Reveals Different Effects of Sulforaphane on the Microbiome and Metabolome in Old Compared to Young Mice. Microorganisms.

[29] Ponce Martínez C., Murcia García E., Pérez Sánchez H., Milagro F.I., Riezu-Boj J.I., Ramos Molina B., Gómez Gallego M., Zamora S., Cañavate Cutillas R., Hernández Morante J.J. (2024). Effect of Silibinin on Human Pancreatic Lipase Inhibition and Gut Microbiota in Healthy Volunteers: A Randomized Controlled Trial. Int. J. Mol. Sci..

[30] Ntemiri A., Ghosh T.S., Gheller M.E., Tran T.T.T., Blum J.E., Pellanda P., Vlckova K., Neto M.C., Howell A., Thalacker-Mercer A. (2020). Whole Blueberry and Isolated Polyphenol-Rich Fractions Modulate Specific Gut Microbes in an In Vitro Colon Model and in a Pilot Study in Human Consumers. Nutrients.

[31] Hou K., Wu Z.X., Chen X.Y., Wang J.Q., Zhang D., Xiao C., Zhu D., Koya J.B., Wei L., Li J. (2022). Microbiota in Health and Diseases. Signal Transduct. Target. Ther..

[32] Qin Y., Zhao J., Wang L., Yang X., Wang J., Li S., Chen Y., Guo J., Wang F., Luo K. (2025). Decrease in Escherichia-Shigella in the Gut Microbiota of ESKD Patients Undergoing Maintenance Hemodialysis. BMC Nephrol..

[33] Chen R., Zhu D., Yang R., Wu Z., Xu N., Chen F., Zhang S., Chen H., Li M., Hou K. (2022). Gut Microbiota Diversity in Middle-Aged and Elderly Patients with End-Stage Diabetic Kidney Disease. Ann. Transl. Med..

[34] Zhang J., Zhao Q., Qin Y., Si W., Zhang H., Zhang J. (2023). The Effect of Epimedium Isopentenyl Flavonoids on the Broiler Gut Health Using Microbiomic and Metabolomic Analyses. Int. J. Mol. Sci..

[35] Kwek E., Yan C., Ding H., Hao W., He Z., Liu J., Ma K.Y., Zhu H., Chen Z.Y. (2022). Effects of Hawthorn Seed Oil on Plasma Cholesterol and Gut Microbiota. Nutr. Metab..

[36] Yan Y., Yuan H., Yang F., Na H., Yu X., Liu J., Wang Y. (2024). Seabuckthorn Polysaccharides Mitigate Hepatic Steatosis by Modulating the Nrf-2/HO-1 Pathway and Gut Microbiota. AMB Express.

[37] Den Besten G., Van Eunen K., Groen A.K., Venema K., Reijngoud D.J., Bakker B.M. (2013). The Role of Short-Chain Fatty Acids in the Interplay between Diet, Gut Microbiota, and Host Energy Metabolism. J. Lipid Res..

[38] Cai H., Su S., Li Y., Zhu Z., Guo J., Zhu Y., Guo S., Qian D., Duan J. (2019). Danshen Can Interact with Intestinal Bacteria from Normal and Chronic Renal Failure Rats. Biomed. Pharmacother..

[39] Xu K.Y., Xia G.H., Lu J.Q., Chen M.X., Zhen X., Wang S., You C., Nie J., Zhou H.W., Yin J. (2017). Impaired Renal Function and Dysbiosis of Gut Microbiota Contribute to Increased Trimethylamine-N-Oxide in Chronic Kidney Disease Patients. Sci. Rep..

[40] Gryp T., Vanholder R., Vaneechoutte M., Glorieux G. (2017). P-Cresyl Sulfate. Toxins.

[41] Wong J., Piceno Y.M., DeSantis T.Z., Pahl M., Andersen G.L., Vaziri N.D. (2014). Expansion of Urease- and Uricase-Containing, Indole- and p-Cresol-Forming and Contraction of Short-Chain Fatty Acid-Producing Intestinal Microbiota in ESRD. Am. J. Nephrol..

[42] Zhu Y., Jameson E., Crosatti M., Schäfer H., Rajakumar K., Bugg T.D.H., Chen Y. (2014). Carnitine Metabolism to Trimethylamine by an Unusual Rieske-Type Oxygenase from Human Microbiota. Proc. Natl. Acad. Sci. USA.

[43] Li G., Li J., Kohl K.D., Yin B., Wei W., Wan X., Zhu B., Zhang Z. (2019). Dietary Shifts Influenced by Livestock Grazing Shape the Gut Microbiota Composition and Co-Occurrence Networks in a Local Rodent Species. J. Anim. Ecol..

[44] Sun P., Wang M., Zheng W., Li S., Zhu X., Chai X., Zhao S. (2023). Unbalanced Diets Enhance the Complexity of Gut Microbial Network but Destabilize Its Stability and Resistance. Stress Biol..

[45] Mougi A., Kondoh M. (2012). Diversity of Interaction Types and Ecological Community Stability. Science.

[46] Coyte K.Z., Schluter J., Foster K.R. (2015). The Ecology of the Microbiome: Networks, Competition, and Stability. Science.

[47] Gryp T., Huys G.R.B., Joossens M., Biesen W.V., Glorieux G., Vaneechoutte M. (2020). Isolation and Quantification of Uremic Toxin Precursor-Generating Gut Bacteria in Chronic Kidney Disease Patients. Int. J. Mol. Sci..

[48] Hayashi T., Yamashita T., Watanabe H., Kami K., Yoshida N., Tabata T., Emoto T., Sasaki N., Mizoguchi T., Irino Y. (2019). Gut Microbiome and Plasma Microbiome-Related Metabolites in Patients with Decompensated and Compensated Heart Failure. Circ. J..

[49] Jing Y., Yang D., Bai F., Wang Q., Zhang C., Yan Y., Li Z., Li Y., Chen Z., Li J. (2023). Spinal Cord Injury-Induced Gut Dysbiosis Influences Neurological Recovery Partly through Short-Chain Fatty Acids. npj Biofilms Microbiomes.

[50] Silva Y.P., Bernardi A., Frozza R.L. (2020). The Role of Short-Chain Fatty Acids From Gut Microbiota in Gut-Brain Communication. Front. Endocrinol..

[51] Shimizu J., Kubota T., Takada E., Takai K., Fujiwara N., Arimitsu N., Ueda Y., Wakisaka S., Suzuki T., Suzuki N. (2016). Bifidobacteria Abundance-Featured Gut Microbiota Compositional Change in Patients with Behcet’s Disease. PLoS ONE.

[52] Hu X., Ouyang S., Xie Y., Gong Z., Du J. (2020). Characterizing the Gut Microbiota in Patients with Chronic Kidney Disease. Postgrad. Med..

[53] Huang Y., Wang Z., Ma H., Ji S., Chen Z., Cui Z., Chen J., Tang S. (2021). Dysbiosis and Implication of the Gut Microbiota in Diabetic Retinopathy. Front. Cell Infect. Microbiol..

[54] Hiippala K., Jouhten H., Ronkainen A., Hartikainen A., Kainulainen V., Jalanka J., Satokari R. (2018). The Potential of Gut Commensals in Reinforcing Intestinal Barrier Function and Alleviating Inflammation. Nutrients.

[55] Palmieri O., Bossa F., Castellana S., Latiano T., Carparelli S., Martino G., Mangoni M., Corritore G., Nardella M., Guerra M. (2024). Deciphering Microbial Composition in Patients with Inflammatory Bowel Disease: Implications for Therapeutic Response to Biologic Agents. Microorganisms.

[56] Taylor W.S. (2021). Prognostic Molecular Markers of Response to Radiotherapy in Rectal Cancer. Master’s Thesis.

[57] Takenaka I.K.T.M., Bartelli T.F., Defelicibus A., Sendoya J.M., Golubicki M., Robbio J., Serpa M.S., Branco G.P., Santos L.B.C., Claro L.C.L. (2022). Exome and Tissue-Associated Microbiota as Predictive Markers of Response to Neoadjuvant Treatment in Locally Advanced Rectal Cancer. Front. Oncol..

[58] Yin L., Xiao X., Georgikou C., Luo Y., Liu L., Gladkich J., Gross W., Herr I. (2019). Sulforaphane Induces MiR135b-5p and Its Target Gene, RASAL2, Thereby Inhibiting the Progression of Pancreatic Cancer. Mol. Ther. Oncolytics.

[59] Lin Q., Dorsett Y., Mirza A., Tremlett H., Piccio L., Longbrake E.E., Choileain S.N., Hafler D.A., Cox L.M., Weiner H.L. (2024). Meta-Analysis Identifies Common Gut Microbiota Associated with Multiple Sclerosis. Genome Med..

[60] Yue M., Jin C., Jiang X., Xue X., Wu N., Li Z., Zhang L. (2023). Causal Effects of Gut Microbiota on Sleep-Related Phenotypes: A Two-Sample Mendelian Randomization Study. Clocks Sleep.

[61] Ntranos A., Park H.J., Wentling M., Tolstikov V., Amatruda M., Inbar B., Kim-Schulze S., Frazier C., Button J., Kiebish M.A. (2022). Bacterial Neurotoxic Metabolites in Multiple Sclerosis Cerebrospinal Fluid and Plasma. Brain.

[62] Lee T.L., Hsuan C.F., Hsu C.C., Wei C.T., Wang C.P., Lu Y.C., Tang W.H., Lu N.H., Chung F.M., Lee Y.J. (2024). Associations of Circulating Total P-Cresylsulfate and Indoxyl Sulfate Concentrations with Central Obesity in Patients with Stable Coronary Artery Disease: Sex-Specific Insights. Int. J. Obes..

[63] He Q., Li G., Zhao J., Zhu H., Mo H., Xiong Z., Zhao Z., Chen J., Ning W. (2024). The Impact of Dysbiosis in Oropharyngeal and Gut Microbiota on Systemic Inflammatory Response and Short-Term Prognosis in Acute Ischemic Stroke with Preceding Infection. Front. Microbiol..

[64] Zhang Q.L., Chen X.H., Zhou S.J., Lei Y.Q., Huang J.S., Chen Q., Cao H. (2023). Relationship between Disorders of the Intestinal Microbiota and Heart Failure in Infants with Congenital Heart Disease. Front. Cell Infect. Microbiol..

[65] Lu Z., Su W., Fan P., Zhu J., Chen C. (2025). Correlation between Indole-3-Acetic Acid and Left Ventricular Hypertrophy in Hemodialysis Patients. Clin. Nephrol..

[66] Nayak S.P.R.R., Boopathi S., Chandrasekar M., Panda S.P., Manikandan K., Chitra V., Almutairi B.O., Arokiyaraj S., Guru A., Arockiaraj J. (2024). Indole-3-Acetic Acid Exposure Leads to Cardiovascular Inflammation and Fibrosis in Chronic Kidney Disease Rat Model. Food Chem. Toxicol..

