# Supplementary material for: Remodeling of Gut Microbial Networks After Sulforaphane Supplementation in Patients with Chronic Kidney Disease
Source: Life (Basel). 2025 Sep 2;15(9):1393. doi: 10.3390/life15091393 (PMC12470981; doi:10.3390/life15091393)
Supplement: Supplementary file 1 [file life-15-01393-s001.zip › life-3835962-supplementary.pdf]

Table S1. co-occurrence networks between the SFN and placebo groups.

Abundant phyla	SFN group (%)		Placebo group (%)	
	Before	After	Before	After
p__Acidobacteriota	1	0	2	2
p__Actinobacteriota	10	5	16	13
p__Bacteroidota	38	32	52	50
p__Bdellovibrionota	1	1	1	1
p__Chloroflexi	2	2	2	2
p__Cyanobacteria	9	7	11	11
p__Desulfobacterota	2	2	2	2
p__Elusimicrobiota	1	1	1	1
p__Firmicutes	40	32	129	52
p__Marinimicrobia_SAR406_clade	1	1	1	1
p__Myxococcota	1	1	1	1
p__Planctomycetota	6	6	7	6
p__Proteobacteria	40	32	52	47
p__Verrucomicrobiota	1	1	4	1
